# Speech Prosody as a Bridge Between Psychopathology and Linguistics: The Case of the Schizophrenia Spectrum

**DOI:** 10.3389/fpsyt.2020.531863

**Published:** 2020-09-15

**Authors:** Valeria Lucarini, Martine Grice, Francesco Cangemi, Juliane T. Zimmermann, Carlo Marchesi, Kai Vogeley, Matteo Tonna

**Affiliations:** ^1^Psychiatry Unit, Department of Medicine and Surgery, Medical Faculty, University of Parma, Parma, Italy; ^2^IfL-Phonetics, University of Cologne, Cologne, Germany; ^3^Department of Psychiatry and Psychotherapy, Medical Faculty, University of Cologne, Cologne, Germany; ^4^Cognitive Neuroscience (INM-3), Institute of Neuroscience and Medicine, Research Center Jülich, Jülich, Germany; ^5^Department of Mental Health, Azienda Unità Sanitaria Locale di Parma, Parma, Italy

**Keywords:** communication, non-emotional, prosody, pragmatics, psychopathology, linguistics, schizophrenia spectrum

## Abstract

Patients with schizophrenia spectrum disorders experience severe difficulties in interpersonal communication, as described by traditional psychopathology and current research on social cognition. From a linguistic perspective, pragmatic abilities are crucial for successful communication. Empirical studies have shown that these abilities are significantly impaired in this group of patients. Prosody, the tone of voice with which words and sentences are pronounced, is one of the most important carriers of pragmatic meaning and can serve a range of functions from linguistic to emotional ones. Most of the existing literature on prosody of patients with schizophrenia spectrum disorders focuses on the expression of emotion, generally showing significant impairments. By contrast, the use of non-emotional prosody in these patients is scarcely investigated. In this paper, we first present a linguistic model to classify prosodic functions. Second, we discuss existing studies on the use of non-emotional prosody in these patients, providing an overview of the state of the art. Third, we delineate possible future lines of research in this field, also taking into account some classical psychopathological assumptions, for both diagnostic and therapeutic purposes.

## Introduction

### Schizophrenia Spectrum and Communication: Disorders of Pragmatic Abilities

Patients with schizophrenia spectrum disorders typically present with significant difficulties in social functioning that can occur in various areas, including in interpersonal communication (1). Schizophrenia has traditionally been described primarily as a communication disorder (2–6). There is currently a great interest in this topic in social cognition research (7).

Successful interpersonal communication relies on conversation partners being able to express and perceive different content *via* their verbal or nonverbal messages. Pragmatics, the branch of linguistics that takes into account the relationship between language and its context, including the correct interpretation of non-literal contents, plays a major role (8). Empirical studies focusing on language have shown a severe impairment of pragmatic abilities (like the capacity to comprehend humor, irony and metaphors) (9, 10) in patients with schizophrenia (11). Moreover, pragmatic deficits negatively correlate with global social functioning (8), significantly contributing, therefore, to the difficulties in social interaction displayed by these patients (12).

Impairments in the comprehension of non-literal meanings, referred to as “concretism” (13), have always been considered distinctive traits of the schizophrenia spectrum by psychopathology. In addition, there is a strong connection between these communication difficulties and one of the core features of the disorders (14, 15), the so-called “hypoattunement” (16, 17) with others, i.e., the incapacity to intuitively grasp unwritten rules of social interactions.

### Prosody as a Fundamental Pragmatic Tool

**Figure 1:**
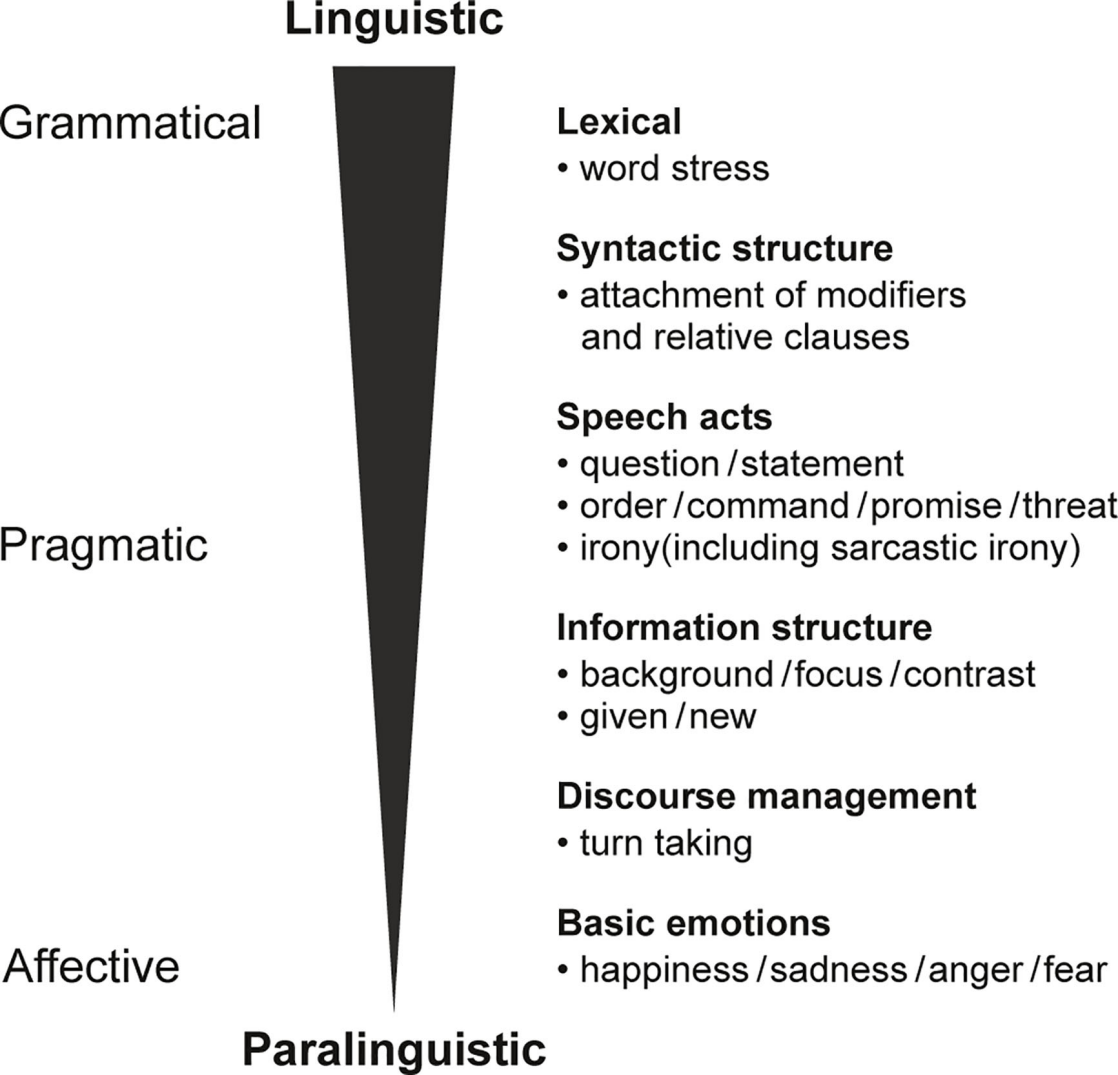
Categorization of prosodic functions [adapted by the authors from Grice and Baumann, (22) and Krüger (18)].

One of the most important carriers of pragmatic meaning is prosody, the tone of voice with which words and sentences are pronounced (18–20). Thus, pragmatic abilities are strongly dependent on prosodic encoding and decoding, achieved mostly through the modulation of fundamental frequency, duration and intensity (18). Prosody is used to divide utterances into chunks, or prosodic phrases, involving the insertion of boundary tones marking the edges of these phrases (18). It also has the role of highlighting certain elements within these phrases by means of accentuation (18). It is important to explore the range of meanings prosody can convey which are often difficult to tease apart and frequently expressed simultaneously. Prosody can have grammatical, pragmatic or emotional functions (21), also referred to as linguistic (grammatical and pragmatic) and paralinguistic (emotional) (22), constituting a continuum (22), as proposed by Grice and Baumann in their model (22) (see **Figure 1**).

At a grammatical level, prosody can provide lexical and syntactic information. For example, in some cases prosody indicates a change in grammatical class (e.g., the word “permit” in English with stress on the first syllable is a noun, while with stress on the second syllable, it is a verb) (18). Prosody can also be used to resolve ambiguities in syntactic structure, such as the attachment of modifiers or relative clauses (e.g., “Jane looked at the man with the binoculars”, in which the binoculars are either used as a viewing device by Jane, or being held by the man being looked at). Prosody can be used to discriminate between questions and statements (18), a function at the interface of grammar and pragmatics. The pragmatic role of prosody can be crucial. In fact, prosody is often the most important means to transmit and understand the communicative purpose of the speaker, helping to distinguish whether a certain phrase is an order, a desire, a promise or a threat. Another important pragmatic role of prosody is the structuring of the elements of a statement in terms of their “givenness”, i.e., whether the element is new or was mentioned before, and therefore given. For example, “I bought a *car*_[NEW]_” refers to the car for the first time (new). A follow-up utterance “Do you want to *see* the car_[GIVEN]_?” refers to the car for a second time and it is therefore given. Referents can also be in focus or in the background (18). For example, in a context such as “What did you buy?” “I bought *a car*_[FOCUS]_”, the car is in focus, whereas in the context “Did you buy a new car?" "No, I *borrowed*[FOCUS] a car[BACKGROUND]” the car is in the background. Moreover, prosody plays a role in controlling turn-taking, e.g., rising pitch indicating the speaker has not yet finished. Finally, prosody can express the emotional state of the speaker (18). Note that such functions (listed in **Figure 1**) are not always clearly distinct and the use of emotional prosody greatly contributes to promoting contextualization in communicative interactions. In recent years, there has been considerable development of technological tools for experimental linguistics, which has permitted the study of these aspects of language in greater depth.

### Research on Prosody in Schizophrenia Spectrum Disorders: State of the Art and Purpose of This Review

Most of the literature on prosody in patients with schizophrenia spectrum disorders has focused on recognition of emotional functions. There is a general consensus on significant impairments in this capacity, despite the heterogeneity of the tasks used (23, 24). However, these deficits only partially explain the difficulties in communicative situations displayed by these patients. Some of the communicative impairments could be further accounted for by difficulties with non-emotional prosody which have scarcely been investigated in patients with schizophrenia spectrum disorders.

The present paper summarizes the main existing studies on the topic. It provides an overview of the state of the art, with papers selected from a search on PubMed and Google Scholar of those published in the period between 1990 and November 2019 (search strategies: schizo* AND prosod*; psychosis AND prosod*; schizophrenia AND prosody NOT emotion NOT affect; psychosis AND prosody NOT emotion NOT affect). From the initial 217 papers, we finally selected 11 studies reporting on patients with any of the following diagnoses: schizophrenia, schizoaffective disorder, first episode psychosis, persons at risk of psychosis, schizotypal personality disorder. We considered all the articles referring to schizophrenia spectrum, together with first episode psychosis and conditions at risk of psychosis, in order to include the whole continuum of different stages of schizophrenia psychopathology (from vulnerability and trait conditions to full-blown disorder). Therefore, the diagnoses of affective psychosis or other psychotic, non-schizophrenia disorders were excluded. We only selected studies in which these groups were compared with healthy controls in their ability to perceive and/or produce non-emotional prosody. Finally, studies had to be written in English. In addition, we manually searched for papers from reference lists of the main articles and reviews, finding one additional study. While focusing mainly on findings regarding pragmatic prosody, we also included results on grammatical prosody, as these are not always clearly distinguishable. Building on this review, the aim of the paper is to identify controversies and limitations of this important, though relatively thin, strand of literature and to delineate possible future lines of research in this field, guided by classical psychopathological notions.

## Perception of Non-Emotional Prosody in Patients With Schizophrenia Spectrum Disorders

Although some studies found an intact performance, there is evidence of a deficit in the perception of non-emotional prosody in patients with schizophrenia spectrum disorders. Below we present the empirical evidence following the continuum of prosodic functions (see **Figure 1**). **Table 1** provides an overview of all studies mentioned in this section.

**Table 1:** Perception of non-emotional prosody by patients with schizophrenia spectrum disorders.

	Grammatical	Pragmatic			
Functions	*Syntactic structure*	*Speech acts*			*Information structure*
Details	Prosodic phrases	Question/Statement	Order/Command	Irony (including sarcastic irony)	Background/Focus/Contrast
**Diff. bt. Groups**	Matsumoto et al. (25) (not sign.)	Pawełczyk et al. (26) Caletti et al. (27)	Pawełczyk et al. (26)	Leitman et al. (28), Kantrowitz et al. (29)	Matsumoto et al. (25)
**No diff. bt. Groups**	Rabagliati et al. (30)	Matsumoto et al. (25) Edwards et al. (31) Castagna et al. (32) Pawełczyk et al. (26, 33) (FEP, UHR, relatives)	Pawełczyk et al. (26, 33) (FEP, UHR, relatives) Caletti et al. (27)		Murphy and Cutting (34)

To our knowledge, no study has assessed the role of prosody to provide lexical information in patients with schizophrenia spectrum disorders so far. The perception of prosody to resolve syntactic ambiguity has been tested by Rabagliati and colleagues (30). In their study, participants with schizophrenia were instructed to manipulate a set of objects on the basis of sentences with variations in the phrasing, determining a bias toward or against a target instrument [e.g., “You can poke the frog … with the feather” vs “You can poke … the frog with the feather” (30)]. The use of the linguistic cues was investigated by tracking eye movements. Results showed that patients and healthy controls did not differ in task performance. This prosodic function was also evaluated by Matsumoto and colleagues (25), who tested patients with schizophrenia and healthy controls in the discrimination of pairs of sentences that differed only in phrasing (like in “Francis, the doctor is ready to begin” and “Francis, the doctor, is ready to begin”) (35, 36). Although patients showed a reduced capacity to recognize these changes, the difference did not reach significance. The same study investigated another prosodic function, namely the discrimination between questions and statements. Sentences (e.g., “She plays the flute”) (35, 36) were pronounced with an intonation indicating either a question or a statement. Patients had to detect and point out the difference. The authors did not find an impairment regarding this ability. These findings were replicated in studies with a similar design, where sentences spoken with statement or question intonation were to be correctly identified. These studies also enrolled patients with first episode schizophrenia (31, 32). Contrary to these findings, Pawełczyk and colleagues (26, 33, 37) recently reported a significant difference between patients with schizophrenia and healthy controls in the use of prosody to decode the communicative purpose of the speaker. They tested patients with schizophrenia, patients with first episode schizophrenia, participants at ultra-high risk of psychosis and first-degree relatives of patients with schizophrenia by means of the “Right Hemisphere Language Battery”. This comprises tasks assessing several pragmatic capacities, including abilities in prosodic processing. Participants listened to sentences read with a statement, question, or command intonation, and indicated for each of them their respective communicative purpose. Apart from the difference between patients with schizophrenia and healthy controls, the authors did not find a difference among subjects at high risk of psychosis, patients with first episode schizophrenia and healthy controls. The ability to detect the same intonation patterns was tested by another recent study (27), which included patients with first episode psychosis (affective and non-affective) and healthy control subjects. Semantically neutral sentences were pronounced with the same three intonation patterns (question/statement/command) and participants had to choose the correct one. The results showed lower scores in both patient groups as compared to controls only regarding the capacity to correctly map question intonation, while no impairment was found for statement and command patterns. Likewise, Leitman and colleagues and Kantrowitz and colleagues (28, 29) investigated the use of prosody to identify speech acts, this time sarcastic irony. They found a deficit in patients with schizophrenia in comparison to healthy controls in the capacity to correctly interpret sentences read in a sincere or sarcastic manner.

Finally, we turn to information structure. Items that are new or in focus are often prosodically highlighted. Murphy and Cutting (34) compared patients with schizophrenia, bipolar disorder in a manic phase, major depression and a group of healthy controls in their ability to recognize the highlighted word in a set of sentences. The authors did not find impairments in patients with schizophrenia. These results conflict with those of Matsumoto and colleagues (25), who also tested patients’ ability to discriminate highlighted words. They used sentence pairs (like “The orange *flowers* smell very sweet” vs “The *orange* flowers smell very sweet”) (35, 36) and found a significant impairment in patients with schizophrenia as compared to controls.

## Production of Non-Emotional Prosody in Patients With Schizophrenia Spectrum Disorders

The production of non-emotional prosody in patients with schizophrenia spectrum disorders has been investigated mainly in terms of acoustic parameters of patients’ speech. Generally, the experiments used to analyze participants’ discourse consisted of clinical interviews (38), free speech tasks (39–43), descriptions of images (44–46) or reading tasks (39, 41, 47). Compared to that of healthy control subjects, the speech of patients on the schizophrenia spectrum appears less fluent (40), contains more and longer pauses (44, 47) as well as less pitch variability (measured as the variance of fundamental frequency for each syllable) (40, 43). Although prosodic parameters were associated neither with antipsychotic dosage (38, 47) nor with positive symptoms (46, 47), an association with negative symptoms was found (38, 40, 46, 48). Moreover, illness-duration had an effect on the performance of patients in prosodic tasks (47). Subjects with schizotypal personality disorder were shown to exhibit a slower speech, with more pauses and less variability in pitch, as compared to healthy controls (41), whereas Cohen and colleagues (45) found differences in prosodic traits only for subjects with negative schizotypal traits.

For the productive use of linguistic prosody, the previously reported study by Murphy and Cutting (34) also tested patients’ ability to highlight a specific word (and thus to indicate its information status). The sentences read aloud by participants were recorded and rated by four raters according to the question which word sounded highlighted to them. The authors did not find a difference between the groups in this task. To our knowledge, only one study, conducted by Michelas and colleagues (49), specifically focused on the production of pragmatic prosody. The authors tested a group of patients with schizophrenia and healthy controls regarding their capacity to signal the focus or background status of an element in a sentence. Participants had to explain to a confederate a designated route on a map, with pairs of landmarks, each composed of two noun-adjective fragments. The pairs could contain the same noun and a different adjective, e.g., bonbons marrons (“brown candies”) vs. bonbons violets (“purple candies”), (49) or a different noun and the same adjective, e.g., bougies violettes (“purple candles”) vs. bonbons violets (“purple candies”) (49). Participants had to use prosodic phrasing to encode the contrastive status of the referent. Even though patients had the ability to produce the same types of phrasing as control participants, they did not appropriately adjust their use of phrasing to the context.

## Discussion

### The Role of Non-Emotional Prosody in Schizophrenia Spectrum: The Evidence So Far

Altogether, there is evidence that in patients with schizophrenia spectrum disorders the capacity for processing grammatical prosody is intact, both with respect to the ability to use phrasing to resolve syntactic ambiguities (30) and with respect to the identification of a question or statement intonation (25, 31, 32), although for the latter there is no general consensus (26, 27). It should be noted that a possible limitation of these studies might be the high simplicity of the tasks, e.g., in (25) patients were required simply to signal if two intonations (question/statement) were different, without having to identify them. When assessing more specifically the prosodic expression of pragmatic functions, these patients show specific impairments as compared to controls (26, 28, 29). In terms of the identification of the speaker’s communicative purpose, the literature focuses on the detection of sarcasm (28, 29) and commands (26). Moreover, patients seem to be impaired in their capacity to use prosody to decode and encode the structural information of a sentence with regard to given/new and focus/background elements (25), although other results conflict with this finding (34). Again, the simplicity of the task of the study of Murphy and Cutting (34) might partially explain the inconsistency of these results.

The use of non-emotional prosody in patients with schizophrenia spectrum disorders has seldom been compared with other clinical groups. Edwards and colleagues (31) did not find significant differences in performance among patients with first episode schizophrenia, first episode affective psychosis or the first episode of other psychotic disorders in their ability to distinguish between a statement and question intonation, similar to the findings of Caletti and colleagues (27). Likewise, in the study of Murphy and Cutting (34), patients diagnosed with schizophrenia, mania, and depression did not differ from the healthy controls regarding their use of pragmatic prosody when recognizing and encoding a highlighted word in a sentence. Few studies investigated a possible association of the perception and production of prosody with clinical measures. Schizophrenia illness duration and antipsychotic treatment dosage have not been shown to correlate with the ability to use prosody to encode the contrastive status of a referent (49) nor with sarcasm detection (29). Results on the relationship with the principal symptom dimensions are controversial. The accuracy to discriminate background/focus information by means of prosodic cues appears negatively correlated with positive symptoms (25), while Michelas and colleagues (49) did not find a relationship between clinical symptomatology and the ability to use prosody to encode the contrastive status of a referent. The ability to detect sarcasm was not associated with positive symptoms, but it correlated with avolition (28). The capacity to correctly map question, statement or command intonation patterns was associated neither with positive symptoms, nor with negative ones (27) in people with first episode psychosis. Altogether, the evidence so far is too scant to draw firm conclusions about these correlations.

Finally, the capacity to use pragmatic prosody was associated with Theory of Mind scores (49) and a significant positive correlation was found between the ability to detect sarcasm and general functioning (29).

In sum, results about the relationship between the use of non-emotional prosody and vulnerability to psychosis (27, 33, 37) are inconclusive. Evidence for impairment in non-emotional prosody processing in first-episode schizophrenia, in ultra-high risk- or in first-degree relative groups was not found, but this last result (33) was not confirmed in larger samples of patients with first episode schizophrenia (27, 37). Interestingly, the Right Hemisphere Language Battery was not originally conceived for patients with schizophrenia. Some tasks may be too simple for less chronically affected patients or unaffected subjects.

The main limitation of the present review is that it is not a systematic one. Nevertheless, to our knowledge this is the first attempt to date to sum up the existing literature about the use of non-emotional prosodic cues by patients with schizophrenia spectrum disorders.

### Perspective on Future Research

The existing literature focusing on the use of non-emotional prosody in patients with schizophrenia spectrum disorders is still very limited. Further research is needed to shed light on the existing results. We suggest that these lines of research should be extended, for a deeper understanding of the specific communicative impairments underlying the disorders. This in turn could contribute to a better diagnosis and possibly help discriminating between schizophrenia and other psychiatric conditions in the future. Moreover, there is evidence of the efficacy of training targeting both pragmatic skills and the use of prosody (50, 51). This could help to design specific and more sophisticated tools, paving the way towards new promising therapeutic approaches.

We have identified some possible points to be addressed by the future research agenda regarding the use of non-emotional prosody by patients with schizophrenia spectrum disorders. The following require investigation:

1. *A number of prosodic functions that have not been investigated so far*. These include (a) The capacity to understand other speakers’ communicative purposes conveyed through prosody, beyond those already tested (sarcasm and commands), especially the ability to correctly detect a threatening disposition. As previously mentioned, the core feature of schizophrenia spectrum is an impairment in the tacit understanding of social situations (17). This can also affect the ability to capture the communicative purpose of the speaker and may elicit compensatory mechanisms (17), contributing to further misinterpretations of social signals, for example leading to persecutory ideas. This is in line with the hypothesis of schizophrenic delusions as due to a “disturbance or breakdown of communication” (52). Another prosodic function is (b) the management of turn-taking. There is evidence of a specific impairment of this function in schizophrenia (53), but the role played by prosody has not been explored so far. A fluid transition in turn-taking implies a high level of rhythmicity between partners (54). A disruption in the shared rhythm between the individual and the environment is traditionally considered a central feature in schizophrenia spectrum (55) and there is empirical evidence for impaired interpersonal synchronization in these patients (56). Prosody, which naturally and implicitly reflects interpersonal synchronization, may represent a key feature of intersubjective “desynchronization” (57) in schizophrenia spectrum disorders. A further prosodic function is (c) the structuring of the elements of a sentence into given/new or focus/background partitions. This has only been scarcely assessed in these patients and a specific investigation of this ability should most definitely be a topic of future research.
2. *Further investigation of the link between prosody deficits and social cognition capacities, such as Theory of Mind*. Schizophrenia has been described as a disorder of social cognition (7) and prosody as a tool playing a crucial role in social interaction (18). A deeper understanding of the use of prosody by patients with schizophrenia spectrum disorders could also shed light on its role in social cognition in general.
3. *The comparison of different clinical groups in their use of non-emotional prosody*. This could help to identify specific profiles of capacities and disorders and to understand if linguistic difficulties (in particular prosodic) are to be considered specific to schizophrenia spectrum disorders. Interestingly, schizophrenia has also been described primarily as a linguistic disorder, (“the price that Homo sapiens paid for language” (Crow, 4). From this perspective, the study of prosody in this clinical population warrants even more interest.
4. *A deeper understanding of possible links between the use of non-emotional prosody and clinical variables, in line with the Research Domain Criteria (RDoC) strategy*. This approach aims at combining several data types, e.g., neurobiological or clinical data, to investigate basic domains of functioning underlying human behavior (like cognition and social processes) for the study of psychiatric conditions (58). For example, investigating if prosodic abilities are linked to negative or positive dimensions could help to understand if linguistic capacities are related to the core symptoms of the disorder.
5. *Further studies assessing the use of non-emotional prosody in people with a vulnerability to schizophrenia*. This would enable us to understand if the impairments are to be considered trait or state conditions. Giving the importance of an early diagnosis in these conditions, it is crucial to find signs that can aid the identification of subjects at risk of schizophrenia prior to the full expression of the disorder.
6. *The examination of the interaction between prosody and other non-verbal cues, like gaze behavior or gestures, on the basis of real-life communicative situations*. To investigate this interaction, it is particularly important to pay attention to the ecological validity of experimental tasks.

A deeper knowledge of the use of non-emotional prosody in patients with schizophrenia spectrum disorders could be helpful also for the study of other communication disorders. Further research should extend this approach to other psychiatric conditions that entail impairments regarding the use of prosody, such as autism spectrum disorders (18, 59, 60).

## Author Contributions

VL wrote the first manuscript version in accordance with theoretical discussions with MG, KV and MT. CM, FC, and JZ contributed with literature and theoretical ideas. All authors read and modified the manuscript several times. All authors contributed to the article and approved the submitted version.

## Funding

The research for this paper has been funded by the German Research Foundation (DFG) as part of the SFB 1252 “Prominence in Language” in project A02 “Individual behavior in encoding and decoding prosodic prominence” at the University of Cologne.

## Conflict of Interest

The authors declare that the research was conducted in the absence of any commercial or financial relationships that could be construed as a potential conflict of interest.
